# Supplementary material for: African Swine Fever Outbreak in an Enclosed Wild Boar Hunting Ground in Serbia
Source: Pathogens. 2023 May 9;12(5):691. doi: 10.3390/pathogens12050691 (PMC10221496; doi:10.3390/pathogens12050691)
Supplement: Supplementary file 1 [file pathogens-12-00691-s001.zip › pathogens-2333604-supplementary.docx]

**Table S1.** Data summary on found wild boar carcasses in the investigated open and enclosed hunting grounds

| **Sample for ASF Detection** | **Geographic Coordinates** | **Sex** | **Age Estimation** | **RT-PCR Result** | **Part of Hunting Ground** | **Estimated PMI** |
| --- | --- | --- | --- | --- | --- | --- |
| Long bone | 45.133615  21.405307 | male | 6 months | + | Open part | 1 |
| Spleen | 45.134621  21.409061 | male | 14 months | + | Open part | 1 |
| Long bone | 45.110335  21.451540 | male | 6 months | + | Open part | 6 |
| Long bone | 45.137214  21.455294 | female | 3 years | + | Open part | 8 |
| Long bone | 45.114610  21.356538 | female | 3 years | + | Open part | 6 |
| Long bone | 45.128435  21.405677 | female | 3 years | + | Open part | 6 |
| Long bone | 45.109044  21.456101 | male | 2 years | + | In the fence | 7 |
| Spleen | 45.110687  21.457185 | male | 6 months | + | In the fence | 1 |
| Long bone | 45.108689  21.454734 | female | 6 months | + | In the fence | 3 |
| Long bone | 45.113892  21.466244 | female | 15 months | + | In the fence | 4 |
| Long bone | 45.116576  21.466028 | female | 3 years | + | In the fence | 12 |
| Long bone | 45.116924  21.450616 | male | 15 months | + | In the fence | 7 |
| Spleen | 45.116872  21.450553 | male | 12 months | + | In the fence | 1 |
| Long bone | 45.118763  21.459017 | female | 4 years | + | In the fence | 8 |
| Spleen | 45.121648  21.477386 | female | 4 years | + | In the fence | 3 |
| Spleen | 45.111921  21.471940 | male | 4 years | + | In the fence | 3 |
| Spleen | 45.108135  21.458347 | male | 2 years | + | In the fence | 2 |
| Long bone | 45.108309  21.459846 | female | 3 years | + | In the fence | 8 |
| Long bone | 45.109930  21.461463 | male | 4 years | + | In the fence | 4 |
| Long bone | 45.109850  21.461439 | male | 6 months | + | In the fence | 8 |
| Long bone | 45.109849  21.461175 | male | 3 months | + | In the fence | 8 |
| Long bone | 45.109540  21.459590 | male | 2 years | + | In the fence | 4 |
| Long bone | 45.122526  21.473777 | female | 3 years | + | In the fence | 4 |
| Spleen | 45.109180  21.454578 | female | 2 years | + | In the fence | 2 |
| Long bone | 45.109471  21.454698 | male | 4 years | + | In the fence | 13 |
| Long bone | 45.109930  21.461463 | female | 3 years | + | In the fence | 3 |
| Spleen | 45.109930  21.461463 | female | 2 years | + | In the fence | 1 |
| Long bone | 45.109930  21.461463 | male | 2 years | + | In the fence | 1 |
| Spleen | 45.109930  21.461463 | male | 2 years | + | In the fence | 3 |
| Long bone | 45.109930  21.461463 | male | 4 years | + | In the fence | 2 |
| Long bone | 45.112970  21.452385 | female | 2 years | + | In the fence | 3 |
| Long bone | 45.112970  21.452385 | female | 6 months | + | In the fence | 4 |
| Long bone | 45.112970  21.452385 | female | 2 years | + | In the fence | 3 |
| Long bone | 45.112970  21.452385 | female | 2 years | + | In the fence | 3 |
| Long bone | 45.117685  21.450672 | female | 3 years | + | In the fence | 4 |
| Long bone | 45.117525  21.450771 | female | 2 years | + | In the fence | 4 |
| Long bone | 45.112332  21.452638 | female | 6 months | + | In the fence | 11 |
| Long bone | 45.112332  21.452638 | female | 1 years | + | In the fence | 8 |
| Long bone | 45.112332  21.452638 | female | 4 years | + | In the fence | 16 |
| Long bone | 45.117679  21.460740 | male | 4 years | + | In the fence | 3 |
| Long bone | 45.120064  21.466587 | male | 6 months | + | In the fence | 6 |
| Long bone | 45.117679  21.460740 | male | 6 months | + | In the fence | 5 |
| Long bone | 45.116499  21.460632 | male | 2 years | + | In the fence | 7 |
| Long bone | 45.116499  21.460632 | male | 1 years | + | In the fence | 3 |
| Long bone | 45.118863  21.467023 | male | 1 years | + | In the fence | 3 |
| Long bone | 45.116499  21.460632 | female | 18 months | + | In the fence | 10 |
| Long bone | 45.118442  21.466790 | male | 3 months | + | In the fence | 3 |
| Long bone | 45.115647  21.460301 | male | 4 years | + | In the fence | 3 |
| Long bone | 45.115647  21.460301 | male | 1 years | + | In the fence | 4 |
| Long bone | 45.114869  21.460189 | male | 4 years | + | In the fence | 3 |
| Long bone | 45.114869  21.460189 | female | 4 years | + | In the fence | 3 |
| Long bone | 45.110128  21.457506 | female | 3 years | + | In the fence | 3 |
| Spleen | 45.110128  21.457506 | male | 1 years | + | In the fence | 2 |
| Long bone | 45.109853  21.456587 | female | 2 years | + | In the fence | 4 |
| Long bone | 45.109853  21.456587 | male | 3 years | + | In the fence | 3 |
| Long bone | 45.109853  21.456587 | female | 1 years | + | In the fence | 4 |
| Long bone | 45.108881  21.455336 | female | 4 years | + | In the fence | 4 |
| Long bone | 45.109516  21.459953 | male | 4 years | + | In the fence | 12 |
| Long bone | 45.109516  21.459953 | male | 4 years | + | In the fence | 5 |
| Long bone | 45.109658  21.460300 | female | 1 years | + | In the fence | 10 |
| Long bone | 45.109959  21.461106 | female | 2 years | + | In the fence | 9 |
| Long bone | 45.109959  21.461106 | female | 2 years | + | In the fence | 8 |
| Spleen | 45.122103  21.448246 | male | 1 years | + | In the fence | 1 |
| Long bone | 45.122103  21.448246 | male | 3 months | + | In the fence | 10 |
| Long bone | 45.110485  21.458132 | male | 3 years | + | In the fence | 3 |
| Spleen | 45.117500  21.450706 | female | 1 years | + | In the fence | 2 |
| Long bone | 45.110485  21.458132 | male | 2 years | + | In the fence | 3 |
| Long bone | 45.117500  21.450706 | male | 1 years | + | In the fence | 2 |
| Long bone | 45.117500  21.450706 | female | 1 years | + | In the fence | 6 |
| Long bone | 45.117500  21.450706 | male | 3 years | + | In the fence | 3 |
| Long bone | 45.110389  21.457329 | male | 2 years | + | In the fence | 2 |
| Long bone | 45.110389  21.457329 | female | 3 years | + | In the fence | 5 |
| Long bone | 45.110389  21.457329 | male | 3 years | + | In the fence | 3 |
| Long bone | 45.110605  21.458195 | female | 3 years | + | In the fence | 2 |
| Long bone | 45.110605  21.458195 | male | 3 years | + | In the fence | 8 |
| Long bone | 45.118329  21.459819 | male | 3 years | + | In the fence | 12 |
| Long bone | 45.118329  21.459819 | male | 3 years | + | In the fence | 9 |
| Long bone | 45.118329  21.459819 | male | 4 years | + | In the fence | 9 |
| Long bone | 45.113552  21.460338 | male | 6 months | + | In the fence | 7 |
| Long bone | 45.113552  21.460338 | male | 2 years | + | In the fence | 7 |
| Long bone | 45.113552  21.460338 | female | 2 years | + | In the fence | 10 |
| Long bone | 45.117168  21.460606 | female | 2 years | + | In the fence | 9 |
| Long bone | 45.117168  21.460606 | male | 1 years | + | In the fence | 12 |
| Long bone | 45.117168  21.460606 | female | 2 years | + | In the fence | 11 |
| Long bone | 45.119979  21.466847 | male | 1 years | + | In the fence | 4 |
| Long bone | 45.119979  21.466847 | female | 2 years | + | In the fence | 2 |
| Long bone | 45.119979  21.466847 | female | 1 years | + | In the fence | 4 |
| Long bone | 45.119979  21.466847 | male | 4 years | + | In the fence | 9 |
| Long bone | 45.119979  21.466847 | male | 2 years | + | In the fence | 3 |
| Long bone | 45.119979  21.466847 | female | 2 years | + | In the fence | 4 |
| Long bone | 45.109930  21.461463 | male | 6 months | + | In the fence | 9 |
| Long bone | 45.109930  21.461463 | female | 2 years | + | In the fence | 9 |
| Long bone | 45.109930  21.461463 | male | 2 years | + | In the fence | 9 |
| Long bone | 45.109930  21.461463 | male | 2 years | + | In the fence | 9 |
| Long bone | 45.119979  21.466847 | female | 6 months | + | In the fence | 7 |
| Long bone | 45.110326  21.457371 | male | 6 months | + | In the fence | 7 |
| Long bone | 45.112727  21.452268 | female | 18 months | + | In the fence | 11 |
| Long bone | 45.112574  21.452587 | male | 30 months | + | In the fence | 6 |
| Long bone | 45.114864  21.473033 | male | 3 years | + | In the fence | 19 |
